# Ap4b1-knockout mouse model of hereditary spastic paraplegia type 47 displays motor dysfunction, aberrant brain morphology and ATG9A mislocalization

**DOI:** 10.1093/braincomms/fcac335

**Published:** 2023-01-06

**Authors:** Joseph M Scarrott, João Alves-Cruzeiro, Paolo M Marchi, Christopher P Webster, Zih-Liang Yang, Evangelia Karyka, Raffaele Marroccella, Ian Coldicott, Hannah Thomas, Mimoun Azzouz

**Affiliations:** Sheffield Institute for Translational Neuroscience (SITraN), Department of Neuroscience, University of Sheffield, Sheffield S10 2HQ, UK; Sheffield Institute for Translational Neuroscience (SITraN), Department of Neuroscience, University of Sheffield, Sheffield S10 2HQ, UK; URI Neuroscience Institute, University of Sheffield, Western Bank, Sheffield S10 2TN, UK; Sheffield Institute for Translational Neuroscience (SITraN), Department of Neuroscience, University of Sheffield, Sheffield S10 2HQ, UK; URI Neuroscience Institute, University of Sheffield, Western Bank, Sheffield S10 2TN, UK; Sheffield Institute for Translational Neuroscience (SITraN), Department of Neuroscience, University of Sheffield, Sheffield S10 2HQ, UK; URI Neuroscience Institute, University of Sheffield, Western Bank, Sheffield S10 2TN, UK; Sheffield Institute for Translational Neuroscience (SITraN), Department of Neuroscience, University of Sheffield, Sheffield S10 2HQ, UK; URI Neuroscience Institute, University of Sheffield, Western Bank, Sheffield S10 2TN, UK; Sheffield Institute for Translational Neuroscience (SITraN), Department of Neuroscience, University of Sheffield, Sheffield S10 2HQ, UK; URI Neuroscience Institute, University of Sheffield, Western Bank, Sheffield S10 2TN, UK; Sheffield Institute for Translational Neuroscience (SITraN), Department of Neuroscience, University of Sheffield, Sheffield S10 2HQ, UK; Sheffield Institute for Translational Neuroscience (SITraN), Department of Neuroscience, University of Sheffield, Sheffield S10 2HQ, UK; URI Neuroscience Institute, University of Sheffield, Western Bank, Sheffield S10 2TN, UK; Sheffield Institute for Translational Neuroscience (SITraN), Department of Neuroscience, University of Sheffield, Sheffield S10 2HQ, UK; URI Neuroscience Institute, University of Sheffield, Western Bank, Sheffield S10 2TN, UK; Sheffield Institute for Translational Neuroscience (SITraN), Department of Neuroscience, University of Sheffield, Sheffield S10 2HQ, UK; URI Neuroscience Institute, University of Sheffield, Western Bank, Sheffield S10 2TN, UK

**Keywords:** hereditary spastic paraplegia, spastic paraplegia type 47, mouse model characterization, pathophysiology

## Abstract

Mutations in any one of the four subunits (ɛ4, β4, μ4 and σ4) comprising the adaptor protein Complex 4 results in a complex form of hereditary spastic paraplegia, often termed adaptor protein Complex 4 deficiency syndrome. Deficits in adaptor protein Complex 4 complex function have been shown to disrupt intracellular trafficking, resulting in a broad phenotypic spectrum encompassing severe intellectual disability and progressive spastic paraplegia of the lower limbs in patients. Here we report the presence of neuropathological hallmarks of adaptor protein Complex 4 deficiency syndrome in a clustered regularly interspaced short palindromic repeats-mediated *Ap4b1*-knockout mouse model. Mice lacking the β4 subunit, and therefore lacking functional adaptor protein Complex 4, have a thin corpus callosum, enlarged lateral ventricles, motor co-ordination deficits, hyperactivity, a hindlimb clasping phenotype associated with neurodegeneration, and an abnormal gait. Analysis of autophagy-related protein 9A (a known cargo of the adaptor protein Complex 4 in these mice shows both upregulation of autophagy-related protein 9A protein levels across multiple tissues, as well as a striking mislocalization of autophagy-related protein 9A from a generalized cytoplasmic distribution to a marked accumulation in the *trans*-Golgi network within cells. This mislocalization is present in mature animals but is also in E15.5 embryonic cortical neurons. Histological examination of brain regions also shows an accumulation of calbindin-positive spheroid aggregates in the deep cerebellar nuclei of adaptor protein Complex 4-deficient mice, at the site of Purkinje cell axonal projections. Taken together, these findings show a definitive link between loss-of-function mutations in murine *Ap4b1* and the development of symptoms consistent with adaptor protein Complex 4 deficiency disease in humans. Furthermore, this study provides strong evidence for the use of this model for further research into the aetiology of adaptor protein Complex 4 deficiency in humans, as well as its use for the development and testing of new therapeutic modalities.

## Teaser

Scarrott *et al*. report a recently generated clustered regularly interspaced short palindromic repeat-mediated mouse model of hereditary spastic paraplegia 47 displays neuroanatomical and behavioural deficits consistent with those observed in human patients and concordant with previous studies in other mouse models of adaptor protein Complex 4 deficiency.

## Graphical Abstract

**Graphical abstract:**
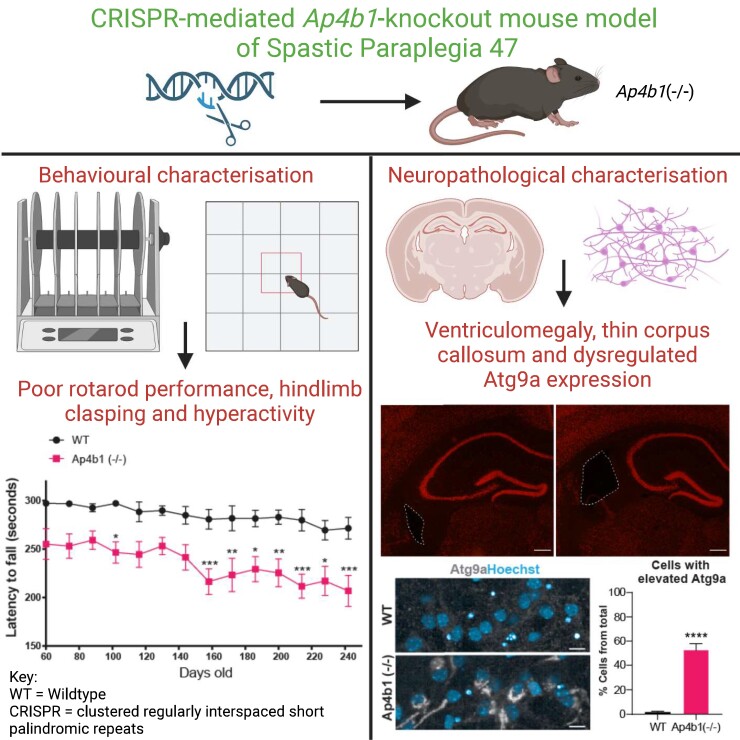

## Introduction

Hereditary spastic paraplegias (HSPs) are a family of progressive lower-limb spasticity disorders characterized pathologically by degeneration of long neuronal axons in the corticospinal and spinocerebellar tracts.^1^ HSPs are both clinically and pathologically heterogenous, whilst the diverse genetic landscape encompasses autosomal dominant, autosomal recessive and X-linked recessive inheritance patterns.^2^ Onset of disease is highly variable, occuring anywhere from infancy to >60 years of age.^3^ HSPs are commonly differentiated into either ‘pure’ or ‘complex/complicated’ subtypes. Pure HSPs are forms in which lower-limb spasticity alone is present, sometimes with other concomitant pathologies. Complex HSP subtypes are generally rare, autosomal recessive and display significant neurological involvement in addition to the progressive lower-limb spasticity of pure HSPs. These neurological components may include—but are not limited to—optic degeneration, white matter degeneration, peripheral neuropathy, intellectual disabilities and epilepsy.^1-4^

Spastic paraplegia Type 47 (SPG47) is a rare HSP subtype caused by autosomal recessive mutations in the ubiquitously expressed Adaptor-related Protein Complex 4 Subunit Beta 1 (*AP4B1)* gene, leading to a significant decrease in AP4B1 protein levels.^5,6^ SPG47 causes initial low muscle tone in infants, which progresses to lower-limb spasticity and muscle weakness. SPG47 is considered a complex HSP, as patients also display numerous cognitive defects including poor speech development and intellectual disability, in addition to loss of movement.^7,8^ Recent work on the underlying biology of SPG47 is strongly indicative of a loss-of-function of the adaptor protein Complex 4 (AP-4).^6,9-11^ AP-4 is a heterotetrameric complex formed of four subunits (ɛ4/β4/µ4/σ4) whose roles include the trafficking of specific cargo proteins between the *trans*-Golgi network (TGN) and the endosome or the nascent autophagosome/phagophore.^10-12^ Loss of any one single AP-4 subunit prevents the correct formation of the AP-4 complex, leading to disruption of protein trafficking and dysregulated localization of the AP-4 cargo protein.^9^ A consistent phenotype observed in cells lacking functional AP-4 is the upregulation of autophagy-related protein 9A (ATG9A) and its mislocalisation in neurons and non-neuronal cells from a diffuse cytoplasmic distribution to a highly conspicuous accumulation within the TGN.^10-13^ AP-4 disruption has also been shown to result in the mislocalisation of a number of somato-dendritic proteins, including AMPA receptors, LDL receptors, transmembrane AMPA receptor regulatory proteins, δ2 glutamate receptors, SERINC1, SERINC3, Sortilin-1 and diacylglycerol lipase-beta.^11,14-17^

In this study, we have generated and characterized a CRISPR-mediated *Ap4b1*-knockout mouse lacking endogenous AP4B1 protein expression, with a particular focus on its suitability for use as a model of AP4-deficient syndromes in human patients. In concordance with previous studies on AP-4-deficient mice,^12-14^ we show that homozygous *Ap4b1*-knockout mice have impaired motor function as determined by rotarod assessment, as well as gait abnormalities, increased incidence of hindlimb clasping characteristic of neurodegeneration, and increased ambulation as assessed by the open-field test. Mice also show neuroanatomical hallmarks of AP-4 deficiency in humans, such as increased ventricular volume and reduced corpus callosum thickness. Immunohistological examination of the CNS of AP4B1-deficient mice reveals mislocalized ATG9A in the cortex, brainstem, hippocampus and deep cerebellar nuclei (DCN), as well as the presence of calbindin-positive spheroids in the DCN, concordant with a previously described AP4E1-KO mouse model.^13^ Mislocalization and overexpression of ATG9A were also found in cortical neurons cultured from E15.5 AP4B1-deficient embryos. Finally, immunoblotting for ATG9A reveals that this protein is overexpressed in the cerebrum, cerebellum, and peripheral tissue of AP4B1-deficient mice, again in concordance with previously published work.^13^

Overall, our study provides strong justification for the use of this murine model for continued investigations into the aetiology of AP-4 deficiency syndromes and for testing and validating new therapeutic modalities, including genetic therapies.

## Materials and methods

### Ethics statement

All animal *in vivo* experiments were approved by the University of Sheffield Ethical Review Sub-Committee, the UK Animal Procedures Committee (London, UK) and performed according to the Animal (Scientific Procedures) Act 1986, under the Project License P31C8CC9D. SOD1^G93A^ mice were maintained in a controlled facility in a 12 h dark/12 h light photocycle (on at 7am/off at 7pm) with free access to food and water. The ARRIVE guidelines have been followed in reporting this study. For behavioural assays and imaging analysis, mice were identified by ID numbers and operators blinded to genotype during assay data collection and analysis. Exclusion criteria for behavioural studies were any animals who were obviously ill or in distress as a result of mutation related phenotype or through general ill-health or injury. No mice were excluded from the analysis due to these criteria. One Ap4b1(−/−) animal in the study was humanely culled at P248 due to sudden illness that was determined to be unrelated to genotype. Data collected from this animal were not excluded from behavioural analyses.

### Genotyping and colony maintenance

C57BL/6J-Ap4b1^em5Lutzy^/J (Strain #: 031349) mice were generated by Jackson Labs using CRISPR-Cas9-mediated deletion of a 76 bp region within Exon 1 of the murine *Ap4b1* gene. Deletion of this region generated a frameshift mutation and a truncated mRNA transcript. WT Sequence (76 bp deletion in lower case):

TTGGCGACGATGCCATAccttggctctgaggacgtggtgaaggaactgaagaaggctctgtgtaaccctcatattcaggctgataggctgcgcTACCGGAATGTCATCCAGCGAGTTATTAGGTATCACCAACCTACCATAGAA.

Genotyping of mice was performed based on the protocol optimized by Charles River Laboratories. Mouse genotyping was performed on genomic DNA extracted from tail or ear tissue by the addition of 20 µl QuickExtract™ DNA Extraction Solution (Lucigen) and incubation on a thermocycler for 15 min at 65°C followed by 2 min at 98°C. Genotyping PCRs were performed in a 20 µl volume reaction as separate reactions for WT and KO alleles. Reactions consisted of 5 µl 5 × FIREPol® Master Mix Ready to Load with 7.5 mM MgCl_2_ (Solis Biodyne), 500 nM each of genotyping primers—GP1 + GP2 for WT allele amplification and GP1 + GP3 for Ap4b1(−/−) allele amplification—(GP1: 5′-TCGCCCGAGGACCCAAGAA—3′; GP2: 5′—CCTATCAGCCTGAATATGAGGGTTACA—3′; GP3: 5′—GCTGGATGACATTCCGGTATATG—3′) and 1 µl genomic DNA from the QuickExtract™ protocol. Touchdown PCR was performed according to the thermal profile shown in Supplementary Table 1. Following PCR and agarose gel electrophoresis (2% agarose gel in Tris-acetate-EDTA buffer), WT and Ap4b1(−/−) allele PCR products were visualized at ∼254 bp and ∼203 bp respectively. Heterozygous mice were bred together to produce homozygous wild-type (WT) (Ap4b1 +/+), Ap4b1-knockout (Ap4b1 −/−) and heterozygous (Ap4b1 +/−) littermates.

### RT-qPCR for human and murine AP4B1 expression analysis.

RT-qPCR was carried out using 2 μl total RNA diluted to a concentration of 10 ng/µl in nuclease free water, 5 µl 2 × QuantiNova SYBR Green RT-PCR Master Mix (Qiagen®), *mAp4b1* (Forward: 5′—CTGTGCTAGGCTCCCACATC—3′; Reverse: 5′—ACGTCCTCAGAGCCAAGGTAT—3′) and *18S* (forward: 5′ GTAACCCGTTGAACCCCAT 3′; reverse: 5′ CCATCCAATCGGTAGTAGCG 3′) primers (all 1 µM concentration), 0.1 µl QN RT mix and H_2_O to a final volume of 10 µl. Following an initial reverse transcription step at 50˚C for 10 min and a 5 min denaturation step at 95˚C, cDNA was amplified by 39 cycles of 95˚C for 10 s followed by a combined annealing/extension step at 60˚C for 10 s. This was followed by one cycle at 65˚C for 31 s, before subsequent melt curve analysis. All RT-qPCR was performed on a Bio-Rad C1000 Touch™ Thermal Cycler. Bio-Rad CFX Manager software was used to analyse signal intensity, and relative gene expression values were determined using the ΔΔCt method, with 18S rRNA used as a reference gene.

### Open field

Open-field analysis was performed on mice [*n* = 16 WT; 16 Ap4b1(−/−); 8 males, 8 females per group) at ages 6, 9 and 12 months. The protocol followed that performed by Herranz-Martin *et al.*.^18^ Mice were placed in a translucent box with dimensions 60 cm × 40 cm × 25 cm. The underside of the box was marked with permanent ink outlining a 5 × 3 grid of squares. Activity was measured as the number of grid lines crossed by each mouse over a 10 min period. For a crossing to be recorded, all four paws of the animal were required to cross the grid line. The assessment was carried out in minimal lighting conditions, and the apparatus was cleaned with 70% ethanol between each animal. One run was recorded for each animal at each timepoint.

### Rotarod

Ugo Basile 7650 accelerating rotarod (set to accelerate from 3–37 rpm over 300 s) was used to measure motor function. Rotarod training was performed over three consecutive days, with two trials per day. Subsequently, this test was performed at bi-weekly intervals in the late morning. For each evaluation, the mice were tested twice, with a minimum rest period of 5 min between runs. The best performance, measured as latency to fall in seconds, was used for analysis. The minimum threshold for recording rotarod activity was 3 s. (N = 16 WT; 16 Ap4b1(−/−); 8 males, 8 females per group)

### Gait analysis

The CatWalk™ gait analysis system version 7.1 was used to assess gait parameters in Ap4b1-KO and WT mice. Mice were tested at 3, 6, 9 and 12 months of age. Mice were placed on the apparatus in complete darkness and their gait patterns recorded. Six unforced runs were recorded for each mouse and three selected for analysis. The runs to be analysed were selected based on the absence of behavioural anomalies—such as sniffing, exploration and rearing—and where mouse locomotion was consistent and without noticeable accelerations, decelerations or deviations from a straight line. Processing of gait data was performed with the Noldus software. Limbs were assigned manually, and gait parameters were calculated automatically. Parameter values were transferred to GraphPad Prism for statistical analysis. (N = 16 WT; 16 Ap4b1(−/−); 8 males, 8 females per group)

### Immunofluorescence staining of mouse brain tissue

Brains were harvested from 8-week-old mice and transferred to 15% sucrose (S24060, Melford) in PBS and subsequently to 30% sucrose. Brains were then frozen using isopentane (Acros Organics, # C5H12) immersed in liquid nitrogen. Sectioning of the brain was performed using a Leica CM3050S cryostat to yield 30 µm-thick coronal slices. ©2004 Allen Mouse Brain Atlas (available from: https://mouse.brain-map.org/static/atlas; Allen Institute for Brain Science) was used to instruct correct investigation of regions of interest in coronal sections (i.e. DCN, hippocampus).^19^ Immunofluorescence staining of coronal sections was performed in free-floating by the following protocol: permeabilization and blocking occurred for 3 h in PBS solution containing 0.1% Triton (Alfa Aesar, # A16046) and 10% Normal goat serum (Abcam, #ab7481). Sections were then incubated with the same solution containing primary antibodies overnight at 4°C. Primary antibodies for immunofluorescence staining of mouse brain tissue included: mouse anti-Calbindin 1:1000 (Abcam, #ab82812); rabbit anti-ATG9A 1:100 (Abcam, #ab108338); rabbit anti-NeuN 1:500 (Cell Signalling, #12943). The secondary antibody AlexaFluor 568 (Thermo Fisher Scientific) was applied in a 1:1000 PBS dilution for 1 hour at room temperature. Washes between each step were done using PBS. Finally, sections were mounted onto charged glass slides (Starfrost, #MBB-0302-55A) in Fluoromount Aqueous Mounting Medium (Sigma, #F4680).

### Haematoxylin/eosin and DAB staining of mouse brain tissue

For haematoxylin/eosin, floating sections were dried on a charged glass slide. Sections were processed according to the following steps: 95% alcohol for 5 min, 70% alcohol for 5 min, H_2_O rinse, haematoxylin (Cellpath, #RBA-4205-00A) solution for 2 min, H_2_O rinse, acid alcohol solution rinse, H_2_O rinse, Scott’s water for up to 60 sec, H_2_O rinse, Eosin solution (Leica, #3801590E) for 5 min, H_2_O rinse, dehydration in alcohols (70%, 90%, 100%) and storage in xylene (Fisher, #X/0200/17). Mounting was then performed with DPX mounting medium (SEA-1300-00A, Cellpath) onto charged glass slides (Starfrost, #MBB-0302-55A). DAB staining was carried out using ABC DAB (Vector Laboratories, #SK-4100) and Elite ABC Kit Vectastain (Vector Laboratories, #PK-6101) according to the manufacturer's instructions. The primary antibody rabbit anti-ATG9A (Abcam, #ab108338) was applied in a 1:100 TBS dilution for 2 h at room temperature. Finally, nuclei were counterstained with Haematoxylin (not shown in Fig. 4) and slides were mounted onto charged glass slides in Fluoromount Aqueous Mounting Medium.

### Statistical analysis

**Figure 1:**
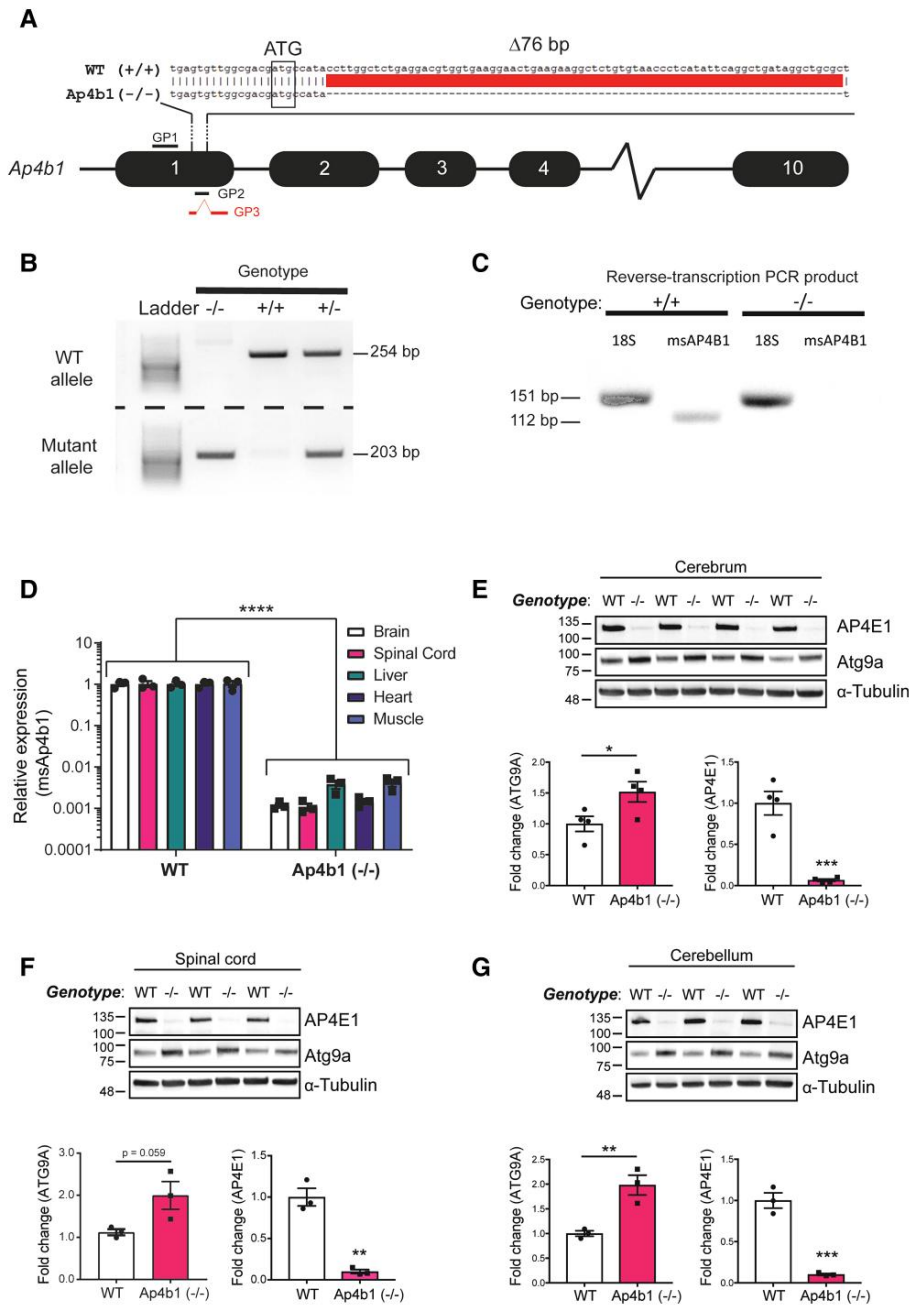
**Genetic characterization of the C57BL/6J-Ap4b1em5Lutzy/J mouse model of SPG47.** Schematic of CRISPR-mediated murine Ap4b1 gene knockout. Guide strand directed DNA cleavage resulted in a 76 bp genomic DNA deletion downstream of the ATG start codon in Exon 1. This deletion is predicted to result in a frameshift leading to a premature stop codon and a non-functional truncated protein. Genotyping primer binding sites are shown as GP1 (forward primer), GP2 (reverse primer), and GP3 (reverse primer) (**A**). PCR-based genotyping of genomic DNA extracted from mouse tail tissue. Two separate PCR reactions generate products for either the WT (254 bp; product from GP1 and GP2 primers) or mutant (Ap4b1 (−/−)) (203 bp; product from GP1 and GP3 primers) allele (**B**). RT-PCR of total RNA extracted from WT (+/+) and Ap4b1 (−/−) mouse brain tissue using primers for murine Ap4b1 (msAP4b1). 18S primers were used as a control (**C**). RT-qPCR of total RNA extracted from both peripheral and CNS tissues showing expression of murine Ap4b1 in Ap4b1(−/−) mice compared to WT mice (**D**). Western blot detection of AP-4 ɛ (AP4E1) and Atg9a protein expression in Ap4b1(−/−) mouse tissue compared to WT, from cerebrum (**E**), spinal cord (**F**) and cerebellum (**G**). Data presented as mean ± S.E.M, analysed by Student’s two-tailed *t*-test (**E–G**) or two-way ANOVA with Bonferroni *post hoc* test for multiple comparisons (D). *n* ≥ 3 animals per group, all animals analysed in this figure were male. *P < 0.05, **P ≤ 0.01, ***P ≤ 0.001, ****P ≤ 0.0001. See Supplementary material (Supplementary Figure 3) for uncropped gel of Fig. 1B. See Supplementary material (Supplementary Figure 4) for uncropped blots of Fig. 1E–G.

**Figure 2:**
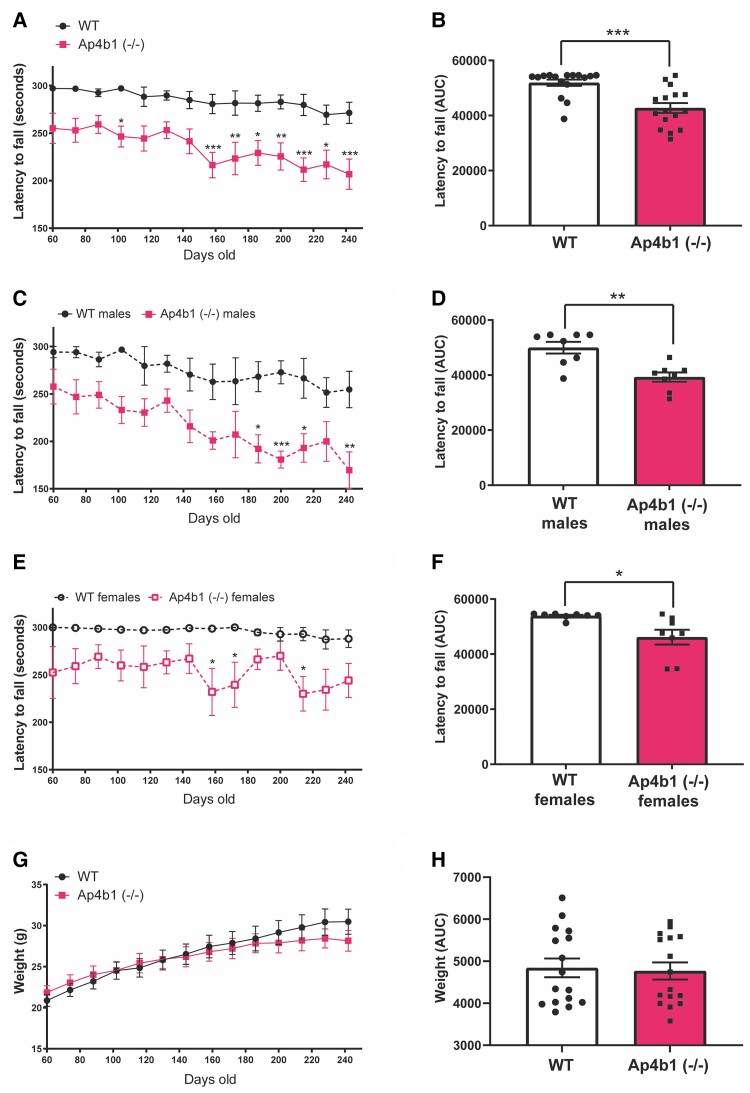
**Ap4b1 (−/−) mouse model of SPG47 displays progressive reduction in motor performance compared with WT mice.** Mice were assessed at several timepoints by accelerating rotarod, with their performance shown as the latency to fall in seconds against age in days (**A**). The area under the curve (AUC) for rotarod performance was calculated and analysed by Student’s two-tailed *t*-test (**B**). Data shown separately for male (**C and D**) and female mice (**E and F**). Total body weight in grams over time was also recorded (**G**). AUC analysis of body weight (**H**). Data are presented as the mean ± S.E.M. Combined sex data *n* = 16 animals per group. Split by sex data *n* = 8 per group. A, C, E and G are analysed by two-way repeated measures ANOVA with Bonferroni *post hoc* test. B, D and F analysed by Student’s two-tailed *t*-test. *P < 0.05, **P ≤ 0.01, ***P ≤ 0.001, ****P ≤ 0.0001.

**Figure 3:**
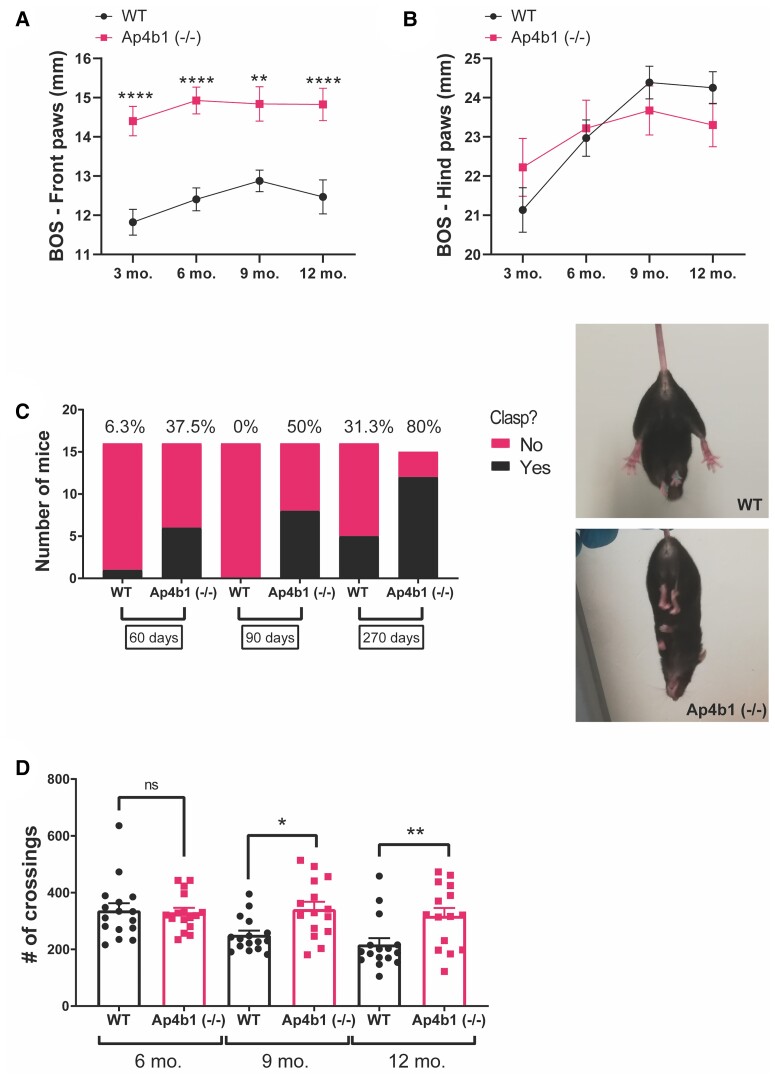
**Behavioural characterization of Ap4b1 (−/−) mice.** Mice were assessed using the Noldus CatWalk gait analysis system at several timepoints. Front paw (**A**) and hind paw (**B**) base of support (BOS) measurements are shown. Percentage of WT and Ap4b1 (−/−) mice who show hindlimb clasping at several timepoints (**C**). Representative non-clasping (WT) and clasping [Ap4b1 (−/−)] images are shown. Open-field analysis of WT and Ap4b1 (−/−) was conducted at several timepoints and shown in (**D**). Data are presented as mean ± S.E.M, *n* ≥ 15 per group; 3 and 6 month timepoints: WT group = 8 males, 8 females; Ap4b1 (−/−) group = 8 males, 8 females. 9 and 12 month timepoints: WT group = 8 males, 8 females; Ap4b1 (−/−) group = 7 males, 8 females. Data were analysed by mixed effects model with Bonferroni *post hoc* test for multiple comparisons. *P < 0.05, **P ≤ 0.01, ****P ≤ 0.0001.

Statistical analysis was performed in GraphPad Prism, version 9.0. Data are presented as mean+/−SEM throughout. Figure 1E–G, 2B, D, F, and H, and Fig. 6B and C were analysed by Student’s two-tailed *t*-test. Statistical significance was determined at *P* < 0.05 for all analyses. Fig. 1D was analysed by two-way ANOVA with Bonferroni *post hoc* multiple comparisons test. One Ap4b1(−/−) animal was humanely culled at P248 due to sudden illness, accordingly a mixed effects model with Bonferroni *post hoc* test for multiple comparisons was performed for CatWalk and open-field analysis in Fig. 3 to take into account the missing data for 9 and 12 month timepoints. In Fig. 5B and C, individual measurements of lateral ventricle area (Fig. 5B) or corpus callosum thickness (Fig. 5C) were normalized within each biological replicate (*n* = 3 per group). Normalized values were then combined and tested for normality using a Shapiro–Wilk test. Owing to lack of normality, mean combined normalized values were then compared using Kolmogorov–Smirnov non-parametric test to detect significant differences. Due to its extremely high value (29-fold greater than replicate normalized value), one data point for the Ap4b1(−/−) group in Fig. 5B has been omitted from the graph (but included in the analysis) to enable clearer data presentation.

### Light microscopy and image analysis

**Figure 4:**
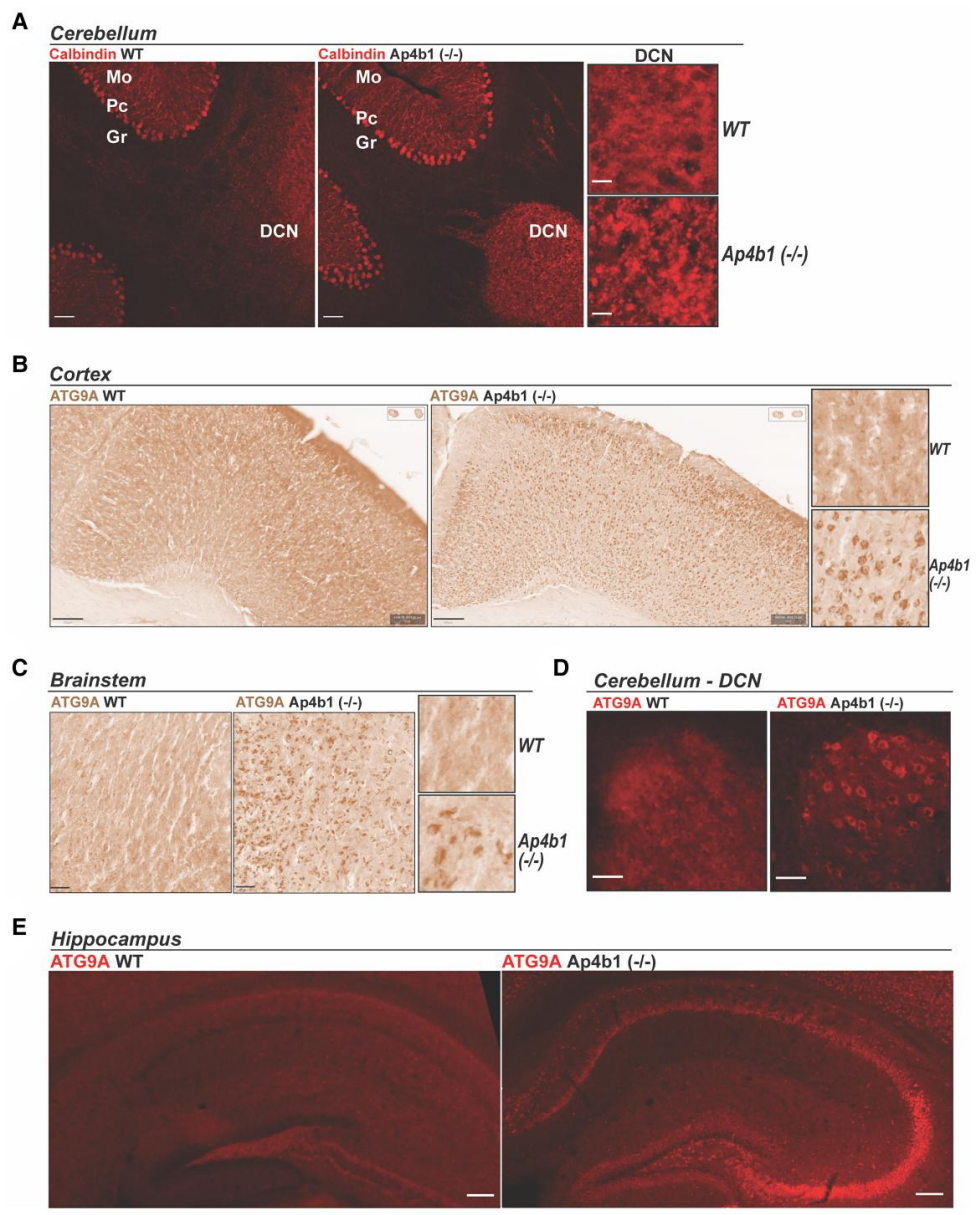
**Histopathological characterization of Ap4b1 (−/−) mice.** Immunohistochemical staining of calbindin in sections from the cerebellum of 8-week-old mice shows characteristic strong staining of Purkinje cell soma in both WT and Ap4b1 (−/−) mice. Additionally, Ap4b1 (−/−) mice but not WT mice display numerous calbindin-positive spheroidal structures in the DCN region where Purkinje cell distal axons terminate (**A**). 3, 3′-diaminobenzidine (DAB) (**B and C**) and immunofluorescent (**D and E**) staining in brain sections from 8-week-old male mice show mislocalized ATG9A protein in cell soma from cortex (**B**), brainstem (**C**), cerebellum (**D**) and hippocampus (**E**) sections from Ap4b1 (−/−) mice. Scale bar: 20 × - 30 µm; 10 × - 50 µm (**A**); 200 µm (**B**); 50 µm (**C**); 20 µm (**D**); 100 µm (**E**).

Immunofluorescence brain sections were imaged using a Nikon Eclipse fluorescence microscope (20 × lens 0.75NA; 10 × lens 0.45NA; 4 × lens 0.20NA; 2 × lens 0.10NA). The 2 × lens was used to image four consecutive NeuN positive-coronal sections (30 µm thickness) for each replicate to study lateral ventricle size. FIJI software was used to calculate the area of the lateral ventricle.^20^ Brain sections stained with Haematoxylin/Eosin or Haematoxylin/DAB were imaged using a Nanozoomer-XR scanner (Hamamatsu). The thickness of the corpus callosum was measured in 15 consecutive Haematoxylin/Eosin-coronal sections (30 µm thickness) for each replicate using the NanoZoomer Digital Pathology software's ruler function. The thickness of the corpus callosum in each section was normalized to the thickness of the entire section. Haematoxylin/DAB sections (for ATG9A) were analysed using QuPath Quantitative Pathology & Bioimage Analysis software.^21^ The Haematoxylin and DAB signals were separated using the QuPath colour transforms function, and ATG9A-DAB positive cells are shown in Fig. 4 after manually drawing a region of interest around the cortex, the brainstem or the hippocampus. Image analysis of brain regions was conducted by an operator blinded to the genotype of each animal.

### Protein extraction and western blotting for protein expression analysis

Tissue was harvested from mice under terminal anaesthesia and snap frozen in liquid nitrogen. Tissue was homogenized using a dounce homogenizer in ice-cold RIPA buffer (50 mM Tris-HCL pH 7.4; 1% v/v NP-40; 0.5% w/v sodium deoxycholate; 0.1% v/v SDS; 150 mM NaCl; 2 mM EDTA) containing 1 × protease inhibitor cocktail (Sigma-Aldrich). Lysate protein concentrations were determined using the BCA assay (Thermo Scientific Pierce™). Protein lysates were denatured by heating to 50°C for 5 minutes in the presence of 4 × laemmli loading buffer (10 ml buffer contained: 240 mM Tris-HCL pH 6.8; 8% w/v SDS; 40% glycerol; 0.01% bromophenol blue; 10% β-mercaptoethanol). Lysates intended to be used for quantification of ATG9A protein levels were not boiled to 100°C as boiling leads to aggregation of ATG9A and loss of signal. Lysates were loaded onto 4–20% gradient mini-PROTEAN® TGX™ precast polyacrylamide gels (Bio-Rad). Gels were run at 150 V in running buffer (25 mM Tris, 192 mM glycine, 0.1% SDS, pH 8.3) for ∼1 h or until the dye front reached the bottom of the gel. Separated proteins were transferred by electrophoresis to 0.2 µm nitrocellulose membrane (Millipore). Protein transfer was carried out at 100 V for 30 min in transfer buffer (25 mM Tris, 192 mM glycine, 20% v/v methanol) using the Bio-Rad Criterion™ blotter. Membranes were blocked for 1 h in 5% milk/TBS-T. Primary antibodies were diluted in 5% milk/TBS-T and incubated with membranes overnight at 4°C. Primary antibodies used in this study for immunoblotting were mouse anti-α-tubulin (1:10000; Sigma #T9026), mouse anti-GAPDH (1:10 000; Millipore #CB1001-500), rabbit anti-ATG9A (1:1000; Abcam #ab108338), mouse anti-AP4E1 (1:1000; BDBiosciences #612019). After primary antibody incubation, membranes were washed for 3 × 10 min in TBS-T buffer. Secondary antibodies anti-mouse HRP (1:5000) and anti-rabbit HRP (1:5000) were diluted in 5% milk/TBS-T and incubated with the membrane for 1 h at room temperature. After secondary antibody incubation, membranes were washed for 3 × 10 min in TBS-T. Protein bands were visualized using ECL Prime Western Blotting Detection Reagent (Amersham) and the G-Box imaging system (Syngene). Densitometric analysis of protein bands was carried out using FIJI software.

### Primary mouse cortical neurons and downstream analyses

E15.5 embryos of C57BL/6 WT and C57BL/6J-Ap4b1^em5Lutzy^/J mice were used to generate primary cortical neurons. Brains were extracted and hemispheres were separated. Meninges and midbrain were removed in HBSS-/− medium to isolate cortical tissue, which was then incubated with trypsin (Gibco) for cell dissociation. Mechanical pipetting in appropriate trituration solution (HBSS+/+ with 1% albumax, 25 mg Trypsin inhibitor, 10 mg/ml DNAse stock) yielded single-cell suspension. Cortical neurons were resuspended in Neurobasal medium (ThermoFisher Scientific) with B27 (Gibco), 1% Pen/Strep (ThermoFisher Scientific) and 1% Glutamine (Lonza) and seeded either on a 96-well optical plates (Greiner Bio-One, #655096) or 12-well tissue culture plates previously coated with poly-D-lysine (Sigma). Cells were kept in a 37°C incubator with 5% CO_2_, and the medium was changed once before the downstream analyses began on Day 7. Cells from the 96-well optical plate were fixed in 4% PFA, permeabilized with 0.2% Triton-X 100:PBS, blocked with 3% BSA and incubated with sheep anti-TGN46 (1:1000; Bio-Rad, #AHP500GT) and rabbit anti-ATG9A (1:1000; Abcam, #ab108338) primary antibodies for 1 h at room temperature. Cells were washed three times in PBS before corresponding Alexa Fluor secondary antibodies and Hoechst (Thermo Fisher Scientific) were applied at a dilution of 1:1000 for 1 h before three final PBS washes. Imaging was performed using Opera Phenix® high-throughput system (PerkinElmer) with 40 × 1.1NA lens. Cells in 12-well plates were directly lysed in 2 × laemmli loading buffer before mechanical shearing of genomic DNA via passage through a 25G needle and 1 ml syringe. Samples were heated to 50°C for 5 min and immunoblotted as described for mouse brain homogenates.

## Results

### Genetic characterization of the C57BL/6j-Ap4b1em5Lutzy/j mouse model of SPG47

Generation of C57Bl/6J mice containing a 76 bp deletion in exon 1 of the murine *Ap4b1* gene [C57BL/6J-Ap4b1em5Lutzy/J; hereafter referred to as Ap4b1 (−/−)] was outsourced to The Jackson Laboratory. The mouse model was generated using guide strand mediated CRISPR nuclease activity (Fig. 1A). Mice for this study were subsequently obtained from Charles River Laboratories (France) after rederivation. Pups were produced by mating heterozygous (+/−) mice, and WT (+/+), homozygous (−/−) and heterozygous offspring were produced at normal Mendellian ratios. WT mice with full-length *Ap4b1* genomic sequence were used as controls in this study. Homozygous (−/−) mice are fertile and capable of generating offspring. Genotyping was performed on DNA extracted from ear-clip tissue using a touchdown polymerase chain reaction (PCR) protocol (Supplementary Table S1). Primers were designed so that a forward primer (GP1) bound 166 bp upstream of the deletion site and two reverse primers recognising either the WT genomic sequence (GP2) or the edited sequence (GP3) (Fig. 1A). Two separate PCR reactions were carried out for the WT and Ap4b1(−/−) alleles, with the resulting agarose gel analysis of the PCR products showing the presence of a single 254 bp band for WT (+/+) mice, a single 203 bp band for homozygous (−/−) mice, and both bands for heterozygous (+/−) mice (Fig. 1B). Reverse-transcription PCR (RT-PCR) of total RNA isolated from brain tissue homogenates using primers designed for murine Ap4b1 (mAp4b1) showed the absence of a product in homozygous knockout mice when analysed by agarose gel electrophoresis (Fig. 1C). In addition, quantitative RT-PCR (qRT-PCR) of total RNA extracted from both CNS and peripheral tissues showed a ∼99% decrease in detectable mAp4b1 expression in Ap4b1(−/−) mice compared to WT mice (Fig. 1D). The 76 bp deletion is likely to result in a frameshift mutation, leading to the presence of a premature stop codon and the formation of a truncated and non-functional protein product. Previous work has demonstrated that human patient fibroblasts harbouring homozygous loss-of-function mutations in the *AP4B1* gene ,^11^ and whole brain lysates from mice harbouring an Ap4b1 null mutation,^14^ also show significant loss of AP4E1 protein expression. In the absence of suitable AP4B1 antibodies, the levels of AP4E1 protein levels were assessed in the brain and spinal cord (Fig. 1E–G) and liver and heart (Supplementary Fig. S1) homogenate from Ap4b1(−/−) mice via western blot. Ap4b1(−/−) mice show significantly reduced expression of the ɛ4 (AP4E1) subunit compared to that of WT mice in all tissues that were analysed. Brain homogenates analysed by western blot also show increased levels of autophagy-related protein 9A (ATG9A) in Ap4b1 (−/−) mice (Fig. 1E–G), as do lysates from peripheral tissues (Supplementary Fig. S1).

### Ap4b1 (−/−) mouse model of SPG47 displays progressive deficits in motor performance compared with WT mice

In patients, SPG47 and other associated AP-4 deficiency syndromes commonly manifest as a progressive motor dysfunction resulting in lower-limb weakness and progressive loss of unassisted ambulation.^22^ In light of this, the motor performance of Ap4b1 (−/−) mice and their WT littermates was assessed by accelerating rotarod. In this test, mice are placed on a rotating cylinder that gradually increases in speed (from 3–37 RPM) over a period of 300 s. The time that each animal is able to remain on the cylinder is measured and recorded. Animals exhibiting deficits in balance and/or muscle strength and co-ordination are unable to remain on the rotarod apparatus at higher speeds and therefore a lower latency to fall value (in seconds) is indicative of reduced motor function. We observed reduced rotarod performance in Ap4b1 (−/−) mice compared with WT mice from the beginning of the rotarod assessment when mice were 60 days old until termination of the assessment at 242 days (Fig. 2A). Disparities in the performance of Ap4b1 (−/−) mice became more pronounced at later timepoints (> 140 days), suggesting a progressive loss of motor function. Area under the curve (AUC) analysis shows a statistically significant reduction in rotarod performance in Ap4b1 (−/−) mice compared with WT littermates (Fig. 2B). Analysis of performance after separating animals by sex shows marked differences between male and female mice. Although the performance deficits in Ap4b1 (−/−) mice compared with WT mice remain apparent in both sexes, rotarod performance in male mice shows a greater decline over time compared with females. Additionally, there is an overall poorer rotarod performance for both genotypes in males (Day 242—WT males: 254.6 s ± 19.3, *n* = 8; Ap4b1 (−/−) males: 169.5 s ± 19.4, *n* = 8) when compared with age-matched females (Day 242—WT females: 288.1 s ± 9.3, *n* = 8; Ap4b1 (−/−) females: 244.1 s ± 17.9, *n* = 8) (Fig. 2C–F). The pattern of rotarod decline in male mice appears progressive, whereas the performance of female mice of both genotypes remains generally stable over the tested period. There were no significant differences in body weight between the two genotypes when analysed together (Fig. 2G and H) or separated by sex (Supplementary Fig. S2) and observed average body weights were typical for C57Bl6/J mice. Despite being often observed clinical manifestations of complex HSPs in patients, no overt ataxia, seizures or epileptic episodes were observed in mice of either genotype.

### Ap4b1 (−/−) mice exhibit hindlimb clasping, increased generalized locomotion and gait abnormalities

CatWalk gait analysis software identified a significantly wider front paw base of support (BOS) in AP-4 β-deficient mice compared with WT mice, which was not observed in the hind limbs and was apparent from as early as 3 months of age (Fig. 3A and B). An increased base of support in rodents has been previously shown to be associated with an unsteady gait, particularly in cases of induced or acquired damage (both traumatic and non-traumatic) to the central nervous system, and correlates with similar gait abnormalities in humans.^23-25^ Although surprising that the gait abnormality was observed in the front paws instead of the hind paws, this may still be indicative of reduced motor co-ordination, as the AP-4 β-deficient mice seek to stabilize an unsteady walking pattern through a widening of their stance. Further evidence of neuromuscular abnormality was the observation that Ap4b1 (−/−) mice suspended by their tails exhibited a distinctive involuntary clasping of the hindlimbs (Fig. 3C). The number of mice exhibiting this clasping phenotype increased as the mice aged, with 80% of Ap4b1 (−/−) mice showing clasping at 270-days-old compared with just under a third of age-matched WT mice (Fig. 3C). Hindlimb clasping has been observed in several other rodent models of neurodegenerative disease, including Amyotrophic Lateral Sclerosis,^26,27^ Rett syndrome,^28^ Leigh disease,^25^ cerebellar ataxia,^29^ Huntington’s disease,^30^ and a previously published model of AP-4 deficiency involving deletion of the ɛ subunit.^13^ Hindlimb clasping has also been associated with degeneration of the spinocerebellar pathway^31^—a clinical hallmark of AP4-deficient patients.^22^ Taken together with the observed front paw gait abnormality, these combined neuromuscular deficits likely account for the reduced rotarod performance of Ap4b1 (−/−) mice in the absence of severe physical manifestations of disease.

Despite the aforementioned motor deficits present in Ap4b1 (−/−) mice, characterization of general locomotor activity via the open-field test showed significantly higher levels of ambulation over the 10 min assay period in Ap4b1 (−/−) mice tested at 9 months [WT: 250.6 crossings ± 15.2, *n* = 16; Ap4b1 (−/−): 341.5 crossings ± 26.2, *n* = 15] and 12 months of age [WT: 216.6 crossings ± 22.9, *n* = 16; Ap4b1 (−/−): 317.5 crossings ± 28.6, *n* = 15], compared with age-matched WT mice (Fig. 3D). Ap4b1 (−/−) mice appeared unable to habituate themselves to an initially novel environment (the open-field apparatus) and exhibited sustained levels of exploratory behaviour (3.6% decrease over 6 months) during subsequent trials, whereas WT mice became accustomed to the apparatus and therefore demonstrated a progressive reduction (35.7% decrease over 6 months) in the number of squares crossed during each subsequent test.

### Histopathological characterization of Ap4b1 (−/−) mice

**Figure 5:**
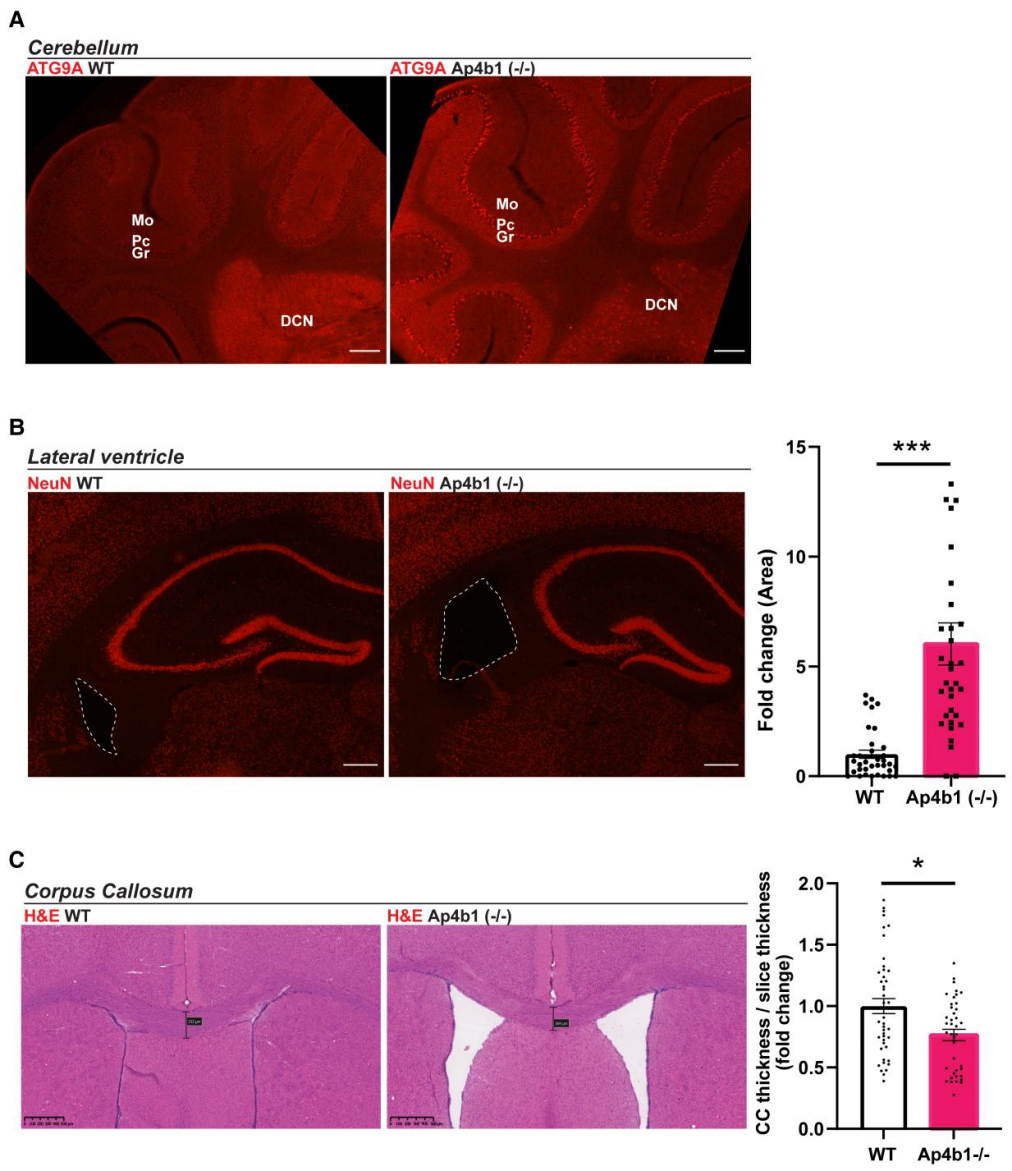
**Mislocalized ATG9A, enlarged lateral ventricles and reduced corpus callosum thickness in the brains of Ap4b1 (−/−) mice.** Immunostaining of ATG9A in cerebellar sections shows mislocalization and increased expression in the Purkinje cell layer and DCN of Ap4b1 (−/−) mice (**A**). NeuN stained coronal brain sections with lateral ventricle highlighted by dashed line in Ap4b1 (−/−) mice. Graph shows quantification of lateral ventricle area as a fold change with respect to WT animals. N = 3, analysed by Kolmogorov–Smirnov test. ***P ≤ 0.001 (**B**). Coronal sections stained with Haematoxylin and eosin (H&E) show reduced corpus callosum thickness in Ap4b1 (−/−) mice. Graph shows quantification of corpus thickness normalized to slice thickness as a fold change with respect to WT animals. Data shown as mean ± S.E.M, *n* = 3, analysed by Kolmogorov–Smirnov test. *P < 0.05 (**C**). All animals analysed were male. Scale bar: 150 µm (A); 500 µm (**C**). Mo = molecular layer, Pc = Purkinje cell layer, Gr = granular cell layer, DCN = deep cerebellar nuclei, H&E = haematoxylin and eosin, ATG9A = autophagy-related protein 9A, CC = corpus callosum.

Neuropathological characterization of various non-AP-4-related spastic paraplegias has previously identified cerebellar abnormalities in a number of complex HSP subtypes.^4,32,33^ Histopathological examination of the cerebellar region of Ap4b1 (−/−) mice did not show any major pathological abnormalities such as atrophy, severe neuronal loss or Purkinje cell loss and the cerebellum appeared morphologically normal compared with WT mice. However immunofluorescent labelling of calbindin, a marker for Purkinje cells, identified unusual calbindin-positive spheroid aggregations in the DCN of Ap4b1 (−/−) mice, the site of axonal projections originating from Purkinje cells (Fig. 4A). Calbindin-positive spheroids have been previously reported in other murine models of AP-4 deficiency.^13,14^ A consistent hallmark of AP-4 deficient cells, and likely the major driver of SPG47 pathology, is the upregulation and mislocalisation of ATG9A, a cargo of the AP-4 complex.^10,11^ ATG9A is usually distributed throughout the cytoplasm but mutations in any of the four AP-4 subunits prevents correct formation of the AP-4 complex, disrupts normal protein cargo trafficking, and leads to ATG9A accumulating at the TGN.^9^ As a result, ATG9A staining in AP-4 β deficient cells displays a characteristic perinuclear pattern due to the accumulation of ATG9A at the TGN and its depletion from the cytoplasm. 3, 3′-diaminobenzidine (DAB)-staining and immunofluorescent staining of ATG9A in coronal tissue sections shows this characteristic staining pattern throughout the brain, including the cortex, brainstem, cerebellar DCN, hippocampus and cerebellum (Fig. 4B–E and Fig. 5A). Staining is particularly strong in the pyramidal neurons comprising the CA3 region of the hippocampus and the large soma of Purkinje cells in the cerebellum.

### Ap4b1 (−/−) mice display neuroanatomical hallmarks of SPG47

In a study of 15 SPG47 patients, 40% displayed ventriculomegaly (enlarged ventricles within the brain) and 73% displayed a thinning of the corpus callosum.^7^ Histological examination of coronal tissue slices from AP-4-deficient mouse brains identified an ∼6-fold increase in lateral ventricle area compared with their WT littermates (Fig. 5B). H&E staining of coronal brain sections allowed measurement of the corpus callosum, which when quantified revealed a significant decrease in corpus callosum thickness in Ap4b1 (−/−) mice compared with WT littermates (Fig. 5C). Ventriculomegaly in humans generally results from either hydrocephalus or from atrophy of brain tissue resulting from neurodegeneration,^34^ and while we do not have definitive confirmation of periventricular tissue atrophy, we did not record any instances of overt hydrocephalus in young mice (i.e. obvious cranial malformation), implying that any possible hydrocephalus occurred after the cranial sutures were closed. Because hydrocephalus at this stage would most likely cause significant pain in the animals, the absence of any distress or increased mortality in older Ap4b1 (−/−) mice would imply that hydrocephalus is not a common occurrence in these animals.

### Primary cortical neurons cultured from E15.5 embryos show ATG9A and AP-4 complex dysregulation

**Figure 6:**
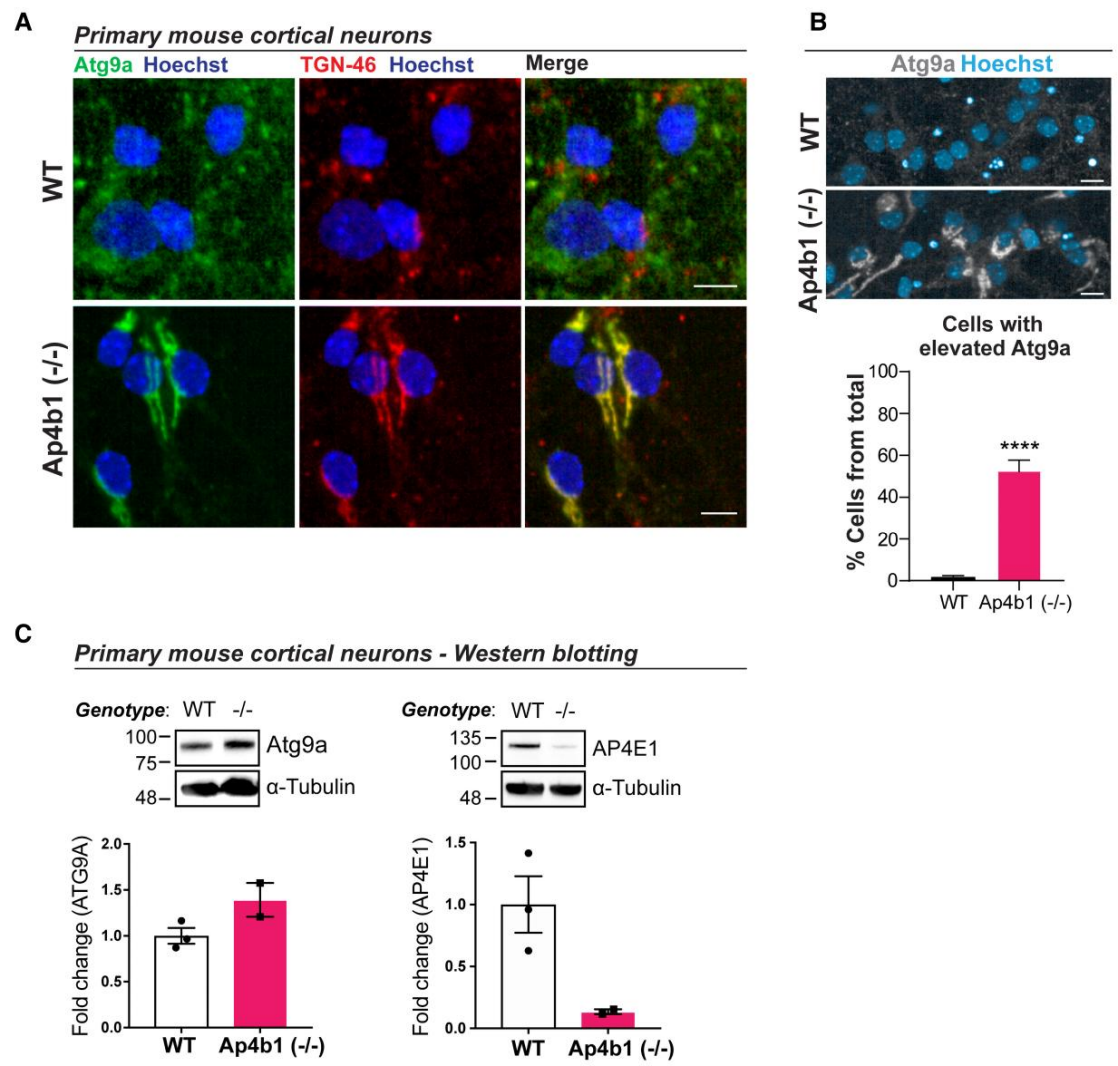
**ATG9A and AP-4 complex dysregulation in primary cortical neurons cultured from embryonic Ap4b1(−/−) mice.** Primary cortical neurons cultured from E15.5 WT or Ap4b1(−/−) embryos were stained with antibodies against Atg9a, a marker for the TGN-46, and Hoescht. Scale bar = 10 µm (**A**). Automated analysis of Atg9a levels in E15.5 cortical neurons. Scale bar = 20 µm (**B**). Western blot analysis of Atg9a and AP4E1 from primary cortical neurons (**C**). *n* = 3 (WT); *n* = 2 [Ap4b1(−/−)]. Data presented as mean+/−SEM and analysed by Student’s unpaired two-tailed *t*-test. ****P ≤ 0.0001. Total number of cells analysed in (B): WT = 9276; Ap4b1(−/−) = 5231 across three (WT) or two (Ap4b1(−/−)) biological replicates. See Supplementary material (Supplementary Figure 5) for uncropped blots of Fig. 6C.

Immunocytochemistry of primary cortical neurons cultured from E15.5 embryos shows mislocalisation of ATG9A to the TGN in cells from Ap4b1 (−/−) embryos (Fig. 6A). Automated analysis of ATG9A levels in primary cortical neurons also shows a significant increase in the number of cells with elevated ATG9A levels in cultures derived from Ap4b1 (−/−) embryos (Fig. 6B). Corresponding levels of these proteins analysed by western blot confirms increased expression of ATG9A whilst also showing reduced AP4E1 expression in cultures derived from Ap4b1(−/−) embryos (Fig. 6C).

## Discussion

SPG47, and AP-4 deficiency syndromes more broadly, manifest in patients as a complex pathology. Multiple symptoms encompassing both neuromuscular and cognitive deficits result in phenotypic heterogeneity even amongst patients with similar genetic mutations.^6,7,22^ Here we show that a murine model of SPG47 recapitulates several known phenotypic hallmarks of AP-4 deficiency.

We conclude from behavioural tests that mice lacking expression of the β subunit of the AP-4 complex exhibit deficits in motor co-ordination and motor performance, hindlimb clasping indicative of neuromuscular degeneration, abnormal gait, and increased overall ambulation. Several of these observations correlate well with symptoms seen in patients suffering from SPG47 and other AP-4 deficiency syndromes.^6,7,35-38^ Deficit in rotarod performance has also been observed in two separately published murine models of AP-4 deficiency.^13,14^ In contrast, the observation of increased ambulation in the open-field test seen in the AP-4 deficient mice does not appear to have a cognate symptom in human patients. This finding has also been described in a previously published murine model of AP-4 deficiency syndrome in which the epsilon subunit of AP-4 was disrupted, suggesting it is a phenotype consistent in mice lacking functional AP-4.^13^ As the Ap4b1 (−/−) mice in this study clearly have reduced motor function—demonstrated by their performance on the rotarod apparatus—we hypothesise that the increased ambulation is related to an undetermined cognitive defect in the mice. Increased locomotor activity in open-field assessments has been associated with hyperactivity and habituation deficits.^39-41^ Habituation deficits in humans have been linked to behavioural problems stemming from hyperactivity disorders which result in a lack of attention.^42,43^ Hyperactivity and inattention as general frequent behavioural problems have been reported in AP-4 associated HSPs,^44^ perhaps offering an explanation for the hyperactive behaviour observed in Ap4b1 (−/−) and other models of AP-4 deficiency syndromes. Alternatively, the increased locomotor activity may serve as a marker for behavioural disinhibition^45,46^ which, although not currently described in SPG47 patients, has been described in other forms of hereditary spastic paraplegia as a consequence of progressive cognitive decline.^47^ We also observe ventriculomegaly in the AP-4-deficient mice in this study, a morphological abnormality that has been shown to affect hippocampal formation and subsequently alter a plethora of cognitive and memory functions in humans.^48,49^ While the observed ventriculomegaly cannot definitively be assigned to periventricular white matter loss (as seen in patients), the absence of cranial malformations suggestive of neonatal hydrocephalus in Ap4b1 (−/−) mice somewhat excludes hydrocephalus as a cause, although CSF volumes were not assessed in the mice. SPG47 patients exhibiting ventriculomegaly are generally clinically diagnosed at a young age, including one case of pre-natal ventriculomegaly,^7^ therefore the ventriculomegaly observed in Ap4b1 (−/−) mice might be a consequence of brain atrophy rather than increased CSF volume. Rats exhibiting spontaneous ventriculomegaly that are otherwise phenotypically unremarkable have impaired memory function leading to a significant reduction in their ability to perform in an object-in-context recognition test.^50^ In one study in humans, 22% of otherwise cognitively normal participants who were identified as having ventriculomegaly were found to perform worse in cognitive tests than those with normal ventricle volume, highlighting ventriculomegaly as an indicator of poor cognitive performance even at sub-clinical levels of cognitive function.^51^ It is likely therefore that the presence of ventriculomegaly in Ap4b1 (−/−) mice is an indicator of impaired cognitive function. De Pace *et al*. (2018) did not find evidence of spatial memory, working memory, or learning abnormalities in AP-4 deficient mice using standard behavioural tests; however, it may be that the tests used were not able to detect subtle changes in cognitive behaviour in the mice.

In all currently characterized murine models of AP-4 complex loss, the overt and severe motor phenotype seen in humans with SPG47 is significantly more mild.^12-14^ The reduced motor phenotype observed in mice compared with humans may be a function of gross anatomical differences related to the proposed mechanism of pathology in affected neurons. In immunohistochemical investigation of numerous CNS locations from Ap4b1 (−/−) mice described here, the retention of ATG9A at the TGN is a distinct pathological hallmark, and is a good diagnostic predictor of AP-4 deficient disorders in patient cells.^52^ As trafficking of ATG9A to distal compartments of neurons is disrupted in AP-4 deficiency syndromes, neurons with longer axons are more likely to be affected by ATG9A deprivation and the resulting reduction in autophagosome biogenesis and distal axon formation.^53-55^ As murine motor neuron axons are shorter than human axons, ATG9A may better reach distal neuronal compartments in the mouse nervous system; for example, via non-AP-4 mediated means—either by incorporation into other AP complexes such as AP-1 and AP-2,^56^ or by passive diffusion.^57^ As a result, ATG9A protein levels in distal axonal compartments in AP-4-deficient mice may be sufficient to prevent severe disease but insufficient to maintain normal autophagic function. Indeed, ATG9A knockout in mice is embryonically lethal or results in death shortly after birth (in conditional knockout models),^58-60^ implying that in AP-4 deficient mice either: loss of correctly localized ATG9A is partially compensated for by other autophagy proteins; or some functional ATG9A protein must be able to reach distal axonal compartments, despite the clear ATG9A trafficking deficits seen in immunohistochemical CNS examination. Low levels of ATG9A protein in distal neuronal compartments may be augmented by the increased expression of ATG9A protein in Ap4b1 (−/−) mice [as shown here by western blot analysis of CNS and peripheral tissue lysates (Fig. 1E–G)], previously hypothesized to be a compensatory mechanism by the cell in an attempt to correct distal ATG9A depletion resulting from AP-4 deficiency.^13^ AP-4 deficient mice display a considerably less severe motor phenotype than SPG47 patients but one that is still significantly different to WT littermates. Severity of disease did appear to slightly differ between sexes—most noticeably in rotarod performance. Although Ap4b1 (−/−) mice of both sexes performed more poorly than their WT littermates, male mice appeared more severely affected in terms of their ability to remain on the apparatus. While we cannot rule out a sex-based difference due to the absence of Ap4b1, we believe that the observed differences can be ascribed to male mice being considerably heavier than females, and therefore less inclined or capable of keeping themselves on the rotating spindle. This difference is most obvious when comparing between WT mice of different sexes; female mice tend to remain on the apparatus for the full 300 s whilst male mice show a steady decline in ability with increasing age (and weight), a discrepancy that has been previously described in rats using the same apparatus.^61^ We do not see sex-based differences in ATG9A expression levels or ATG9A distribution in tissue.

Our observation of pre-natal ATG9A mislocalization in cortical neurons cultured from E15.5 Ap4b1(−/−) embryos leads us to suspect that mice lacking AP-4 function may already be born with defective autophagic function. We believe that the reduced corpus callosum thickness and increased ventricular volume may therefore result from a combination of neurodevelopmental defects, poor and impaired neuronal outgrowth and subsequent progressive neuronal degeneration, rather than exclusively neuronal loss occurring postnatally. Although the progressive motor phenotype observed in human subjects is not as apparent in the murine model (theories of which are discussed above), the neurodevelopmental phenotype seen here, in pre-natal observations in patients,^44^ and in observations made for other early-onset neurodegenerative diseases,^62^ forcefully highlight the need for early diagnoses and therapeutic intervention to maximise beneficial patient outcomes. These findings also raise the possibility that some symptoms stemming from neurodevelopmental defects occurring prenatally may not be amenable to treatment or rescue, depending on the extent of neuronal damage or loss at birth and the difficulty in initiating early treatment due to a combination of low disease incidence and phenotypic overlap with higher incidence disorders (such as cerebral palsy, other early-onset spastic paraplegias and unrelated inborn errors of metabolism^22^) resulting in delayed diagnosis.

In summary, we show here that a CRISPR-mediated Ap4b1-knockout murine model of human hereditary spastic paraplegia 47 exhibits both behavioural and neuroanatomical deficits that in part mirror those seen in patients. We believe this to be a comprehensive study with immediate utility for translational studies, which builds on previous work linking nonsense mutations in *Ap4b1* in mice to symptoms that are representative of human AP-4 deficiency syndrome. Most strikingly this model reproduces ATG9A mislocalization, upregulation, and accumulation across multiple brain regions within the CNS which is a robust indicator of AP-4 deficiency and disease. The various readouts presented here will enable the model to be used for drug discovery, putative gene therapies, or testing of new therapeutic modalities to ameliorate disease in patients suffering from not just SPG47, but for all AP-4 deficiency diseases linked by their shared pathological phenotypes. It will also enable further investigation of the biochemical drivers of HSP47 and contribute to a greater understanding of the complex interactions between genotype and phenotype that is a hallmark of AP-4 deficiency syndromes.

## Supplementary Material

fcac335_Supplementary_Data

## Data Availability

Data are available by direct request to authors.

## Abbreviations

AP-1 =: adaptor protein complex-1
AP-2 =: adaptor protein complex-2
AP-4 =: adaptor protein Complex-4
AP4B1 =: adaptor-related protein Complex 4 Subunit Beta 1
AP4E1 =: adaptor-related protein Complex 4 subunit epsilon 1
ATG9A =: autophagy-related protein 9A
BOS =: base of support
DAB =: 3, 3'-diaminobenzidine
DCN =: deep cerebellar nuclei
HSP =: hereditary spastic paraplegia
PFA =: paraformaldehyde
qRT-PCR =: quantitative reverse-transcription PCR
RIPA =: radioimmunoprecipitation buffer
RT-PCR =: reverse-transcription PCR
SPG47 =: spastic paraplegia type 47
TGN =: *trans*-Golgi network
WT =: wild-type
