# Supplementary material for: Ap4b1-knockout mouse model of hereditary spastic paraplegia type 47 displays motor dysfunction, aberrant brain morphology and ATG9A mislocalization
Source: Brain Commun. 2023 Jan 6;5(1):fcac335. doi: 10.1093/braincomms/fcac335 (PMC9825813; doi:10.1093/braincomms/fcac335)
Supplement: fcac335_Supplementary_Data [file fcac335_supplementary_data.docx]

Supplementary figures and tables

| *Supplementary Table 1 – Touchdown PCR conditions for C57BL/6J-Ap4b1em5Lutzy/J genotyping* | | | |
| --- | --- | --- | --- |
| **Step** | **Temp (°C)** | **Time (sec)** | **Number of Cycles** |
| Initial Denaturation | 94 | 120 | 1 |
| Denaturation | 94 | 60 | 10 cycles |
| Annealing | TD 58 -54 | 60 |  |
| Elongation | 72 | 60 |  |
| Denaturation | 94 | 60 | 25 cycles |
| Annealing | 54 | 60 |  |
| Elongation | 72 | 60 |  |
| Final Elongation | 72 | 300 | 1 |


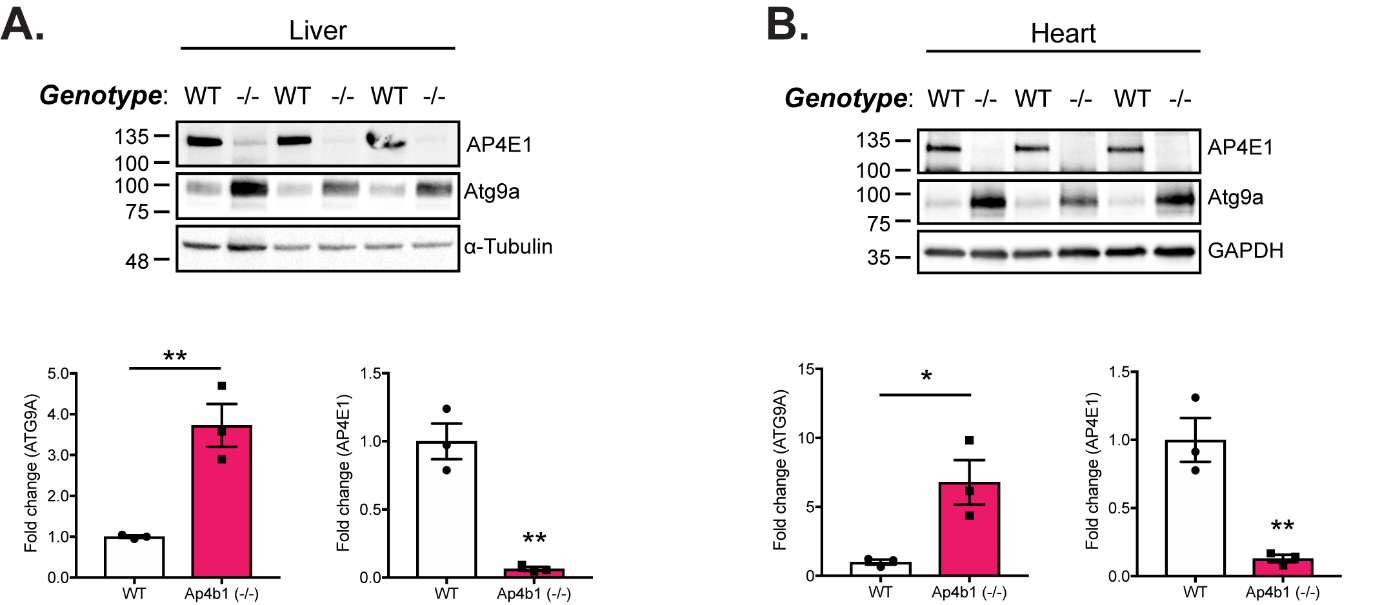


**Supplementary Figure 1 –** Western blot analyses of Atg9a and AP4E1 protein levels in peripheral tissues. Data presented as mean ± S.E.M, n = 3 animals per group, analysed by Student’s two-tailed t-test. *p < 0.05, **p **≤** 0.01.


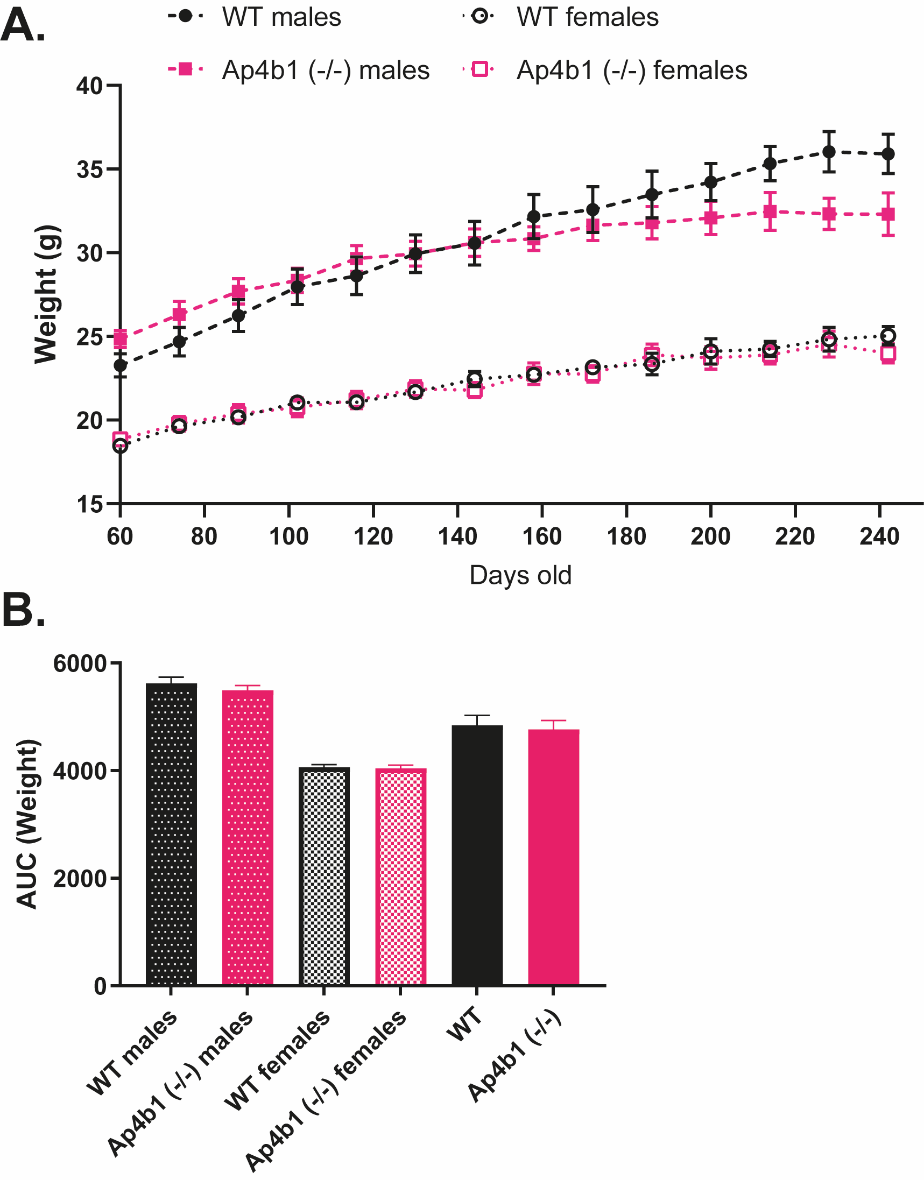


**Supplementary Figure 2** – Weight measurements for WT and Ap4b1 (-/-) mice split by sex (**A**). Area under the curve (AUC) values for weight presented as separate sex and combined sex (solid bars) (**B**). Data presented as mean ± S.E.M, n = 8 males and 8 females per group (combined data: n = 16).


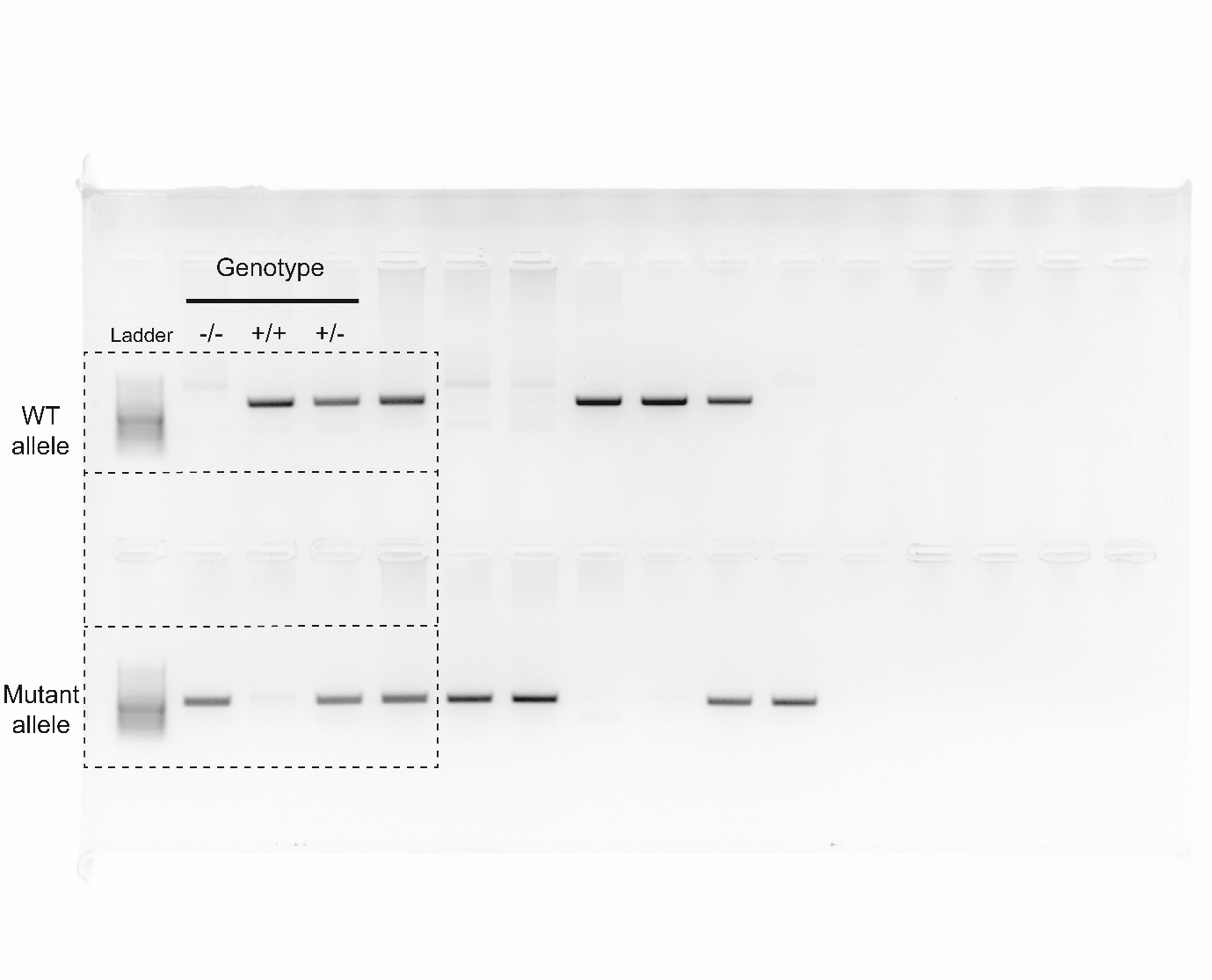


**Supplementary figure 3 – Uncropped gel image of Figure 1B.**


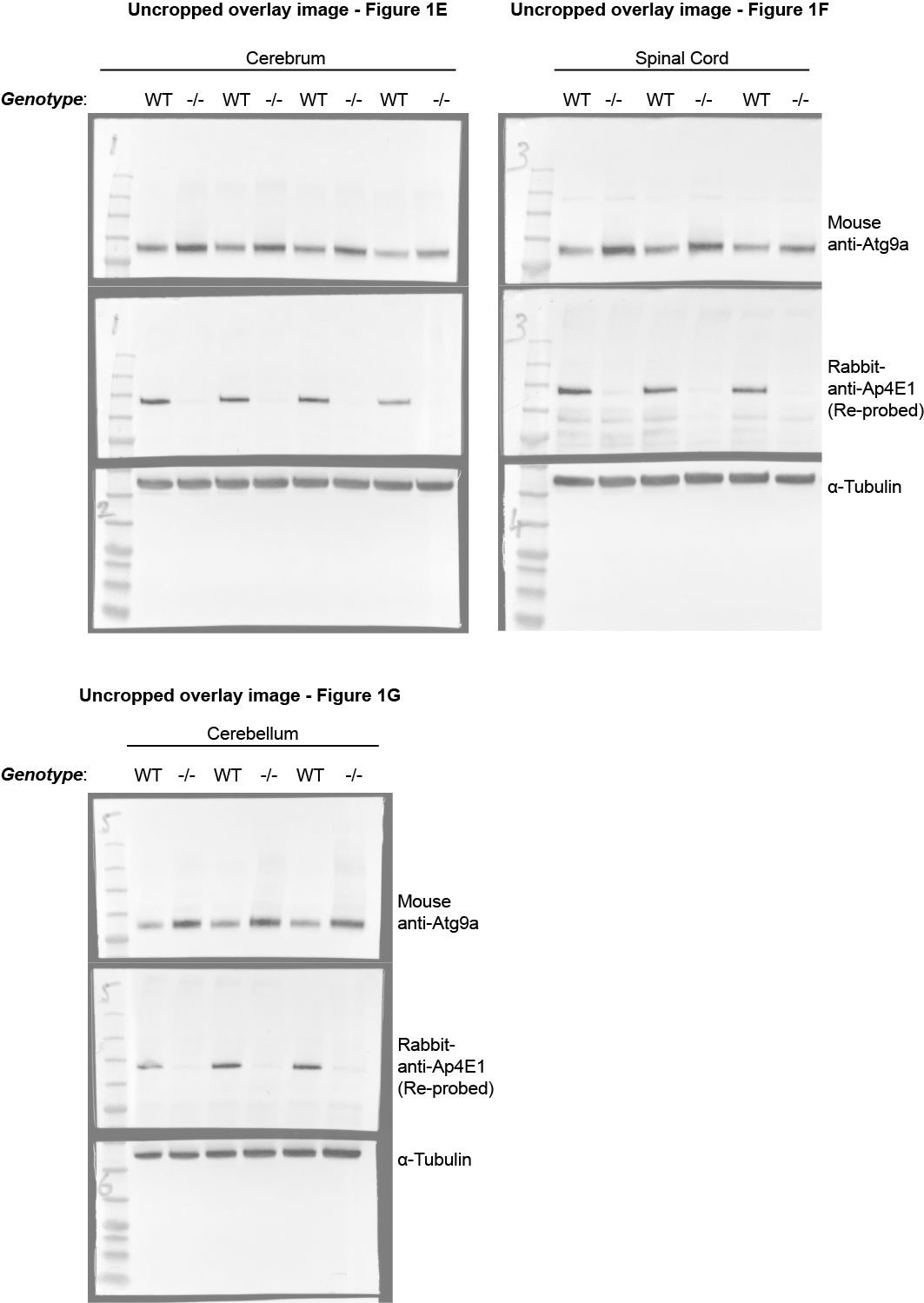


**Supplementary figure 4 – Uncropped Western blot images from Figure 1E -G.**

**
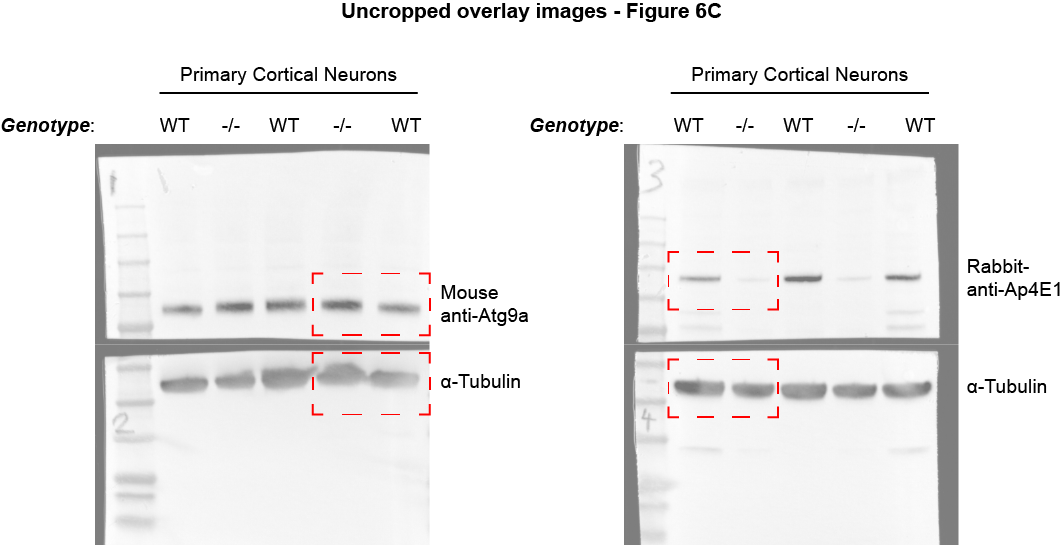
**

**Supplementary figure 5 – Uncropped Western blot images from Fig 6C.**
